# Biochar Coating as a Cost-Effective Delivery Approach to Promoting Seed Quality, Rice Germination, and Seedling Establishment

**DOI:** 10.3390/plants12223896

**Published:** 2023-11-18

**Authors:** Kangkang Zhang, Xiaomeng Han, Yanfeng Fu, Yu Zhou, Zaid Khan, Junguo Bi, Liyong Hu, Lijun Luo

**Affiliations:** 1 National Key Laboratory of Crop Genetic Improvement, College of Plant Science and Technology, Huazhong Agricultural University, Wuhan 430070, China; zhangkk@webmail.hzau.edu.cn (K.Z.); hanxiaomeng@webmail.hzau.edu.cn (X.H.); fuyanfeng@webmail.hzau.edu.cn (Y.F.); zy2306@webmail.hzau.edu.cn (Y.Z.); 2 Key Laboratory of Grain Crop Genetic Resources Evaluation and Utilization, Ministry of Agriculture and Rural Affairs, Shanghai 201106, China; 3 Shanghai Agrobiological Gene Center, Shanghai 201106, China; 4 College of Natural Resources and Environment, South China Agricultural University, Guangzhou 510642, China; zaidkhan@scau.edu.cn

**Keywords:** biochar, seed coating, seed quality, water-saving and drought-resistance rice (WDR), seed water absorption, respiration rate

## Abstract

The application of high-quality seeds ensures successful crop establishment, healthy growth, and improved production in both quantity and quality. Recently, biochar-based seed coating has been recognized as a new, effective, and environmentally friendly method to enhance seed quality, seedling uniformity, and nutrient availability. To study the impact of biochar coating on the surface mechanical properties of coated seeds, rice emergence and growth, and related physical and physiological metabolic events, laboratory experiments were performed on two water-saving and drought-resistance rice (WDR) varieties (Huhan1512 and Hanyou73) using biochar formulations with varying contents (20%–60%). The results showed that the appropriate concentration of biochar significantly improved emergence traits and seedling performance of the two rice varieties, compared to the uncoated treatment, and that the optimal percentage of biochar coating was 30% (BC30). On average, across both varieties, BC30 enhanced emergence rate (9.5%), emergence index (42.9%), shoot length (19.5%), root length (23.7%), shoot dry weight (25.1%), and root dry weight (49.8%). The improved germination characteristics and vigorous seedling growth induced by biochar coating were strongly associated with higher water uptake by seeds, increased α-amylase activity and respiration rate, and enhanced accumulation of soluble sugar and soluble protein. Moreover, the evaluation results of mechanical properties related to seed coating quality found that increasing the proportion of biochar in the coating blend decreased the integrity and compressive strength of the coated seeds and reduced the time required for coating disintegration. In conclusion, biochar coating is a cost-effective strategy for enhancing crop seed quality and seedling establishment.

## 1. Introduction

Seeds are agricultural products with high added value and represent not only a food source but also the most important resource in agricultural practice [1]. Farmers commonly demand high-quality seeds of diverse crop varieties, as they can guarantee food security by promoting crop production [2]. Moreover, quality seeds with high vigor can achieve uniform emergence and successful stand establishment, potentially increasing crop outputs by more than 30% [3,4]. Therefore, it is necessary to obtain and enhance seed quality to improve agricultural productivity. However, numerous limitations negatively impact seed quality and subsequent seedling performance, including seed hardness and aging, soil-borne diseases, and adverse environmental challenges [5,6]. The use of precise tools to improve seed characteristics and value and support germination is paramount to guarantee efficiency in terms of grain yield.

Seed coating is a promising technique aimed at increasing seed quality and performance through the application of suitable seed coating agents with appropriate contents [7,8]. Coating agents mainly include a binder (adhesive substance), a filler (bulking agent), and active ingredients (e.g., nutrients, protectants, and inoculants) [8,9]. Active ingredients serve to enhance and protect seed and seedling performance, encompassing aspects such as emergence, growth, and development. Studies have shown that seed treatment can lead to improved germination and plant growth when compared to untreated seeds, requiring even lower amounts of active ingredient per hectare than foliar application due to the decreased surface area after treatment [10]. Nevertheless, there is still a lack of organic and safe coating materials that are effective in stimulating seed sprouting and plant growth [9,11].

Biochar is expected to be a desirable and promising candidate for seed coating. Biochar is an environmentally persistent organic material which is rich in carbon and obtained by pyrolyzing biomass at high temperatures under limited oxygen conditions [12]. Due to the large variation in feedstocks and pyrolysis conditions, biochar can have various physical and chemical characteristics such as porous structures, rich nutrients, large surface area, higher organic carbon content, moisture-holding capacity, etc. [9]. Previous studies have documented that biochar includes smoke-derived substances and growth-promoting compounds, which emit potent chemical signals, facilitating emergence and plant establishment via the enhancement of soil texture and moisture retention ability [13,14]. Furthermore, evidence from field trials has confirmed that biochar as a seed coating agent is favorable for improving rice emergence rate and stand establishment, ultimately maximizing grain yield [9,11] reviewed the literature extensively and concluded that the promotion of stand establishment may be due to the high porosity and large surface area of biochar, which enhances water availability and rich nutrients around the seeds.

Recently, water-saving and drought-resistant rice (WDR) varieties have been widely grown by farmers in China due to their high yield potential and great grain quality. These varieties have superior traits such as high water-use efficiency and drought tolerance and can be sown not only in paddy fields but also in rainfed fields. In order to ensure proper germination and growth in drylands, it is necessary to apply coating techniques to treat WDR seeds and improve seed vigor. However, previously, very little information was available on the potential of combining WDR seeds with a seed coating using biochar. It is well recognized that the physical properties and thickness of the seed coating are the most significant factors affecting seed germination and plant performance. For example, a hard and thick coating may reduce or delay germination or even cause mortality. Conversely, a minimal and fragile coating could disintegrate before sowing or not be effective due to a lack of active ingredients. Therefore, specialized seed coating formulations must be developed and evaluated for effective use in rice production. The objectives of this study were to (1) screen the optimum biochar coating formulations based on the physical properties and performance of coated rice seeds and (2) reveal the underlying physical, physiological, and biochemical mechanisms of biochar coating to promote rice germination and seedling growth.

## 2. Materials and Methods

### 2.1. Seed and Coating Materials

The seeds employed in this investigation consisted of Hanyou73 (HY73), characterized as a hybrid WDR variety, and Huhan1512 (HH1512), classified as a conventional WDR variety. Both varieties were developed by the Shanghai Agrobiological Gene Center. Biochar (BC), a coating agent frequently employed in various research endeavors, was derived through the pyrolysis of rice straw at an elevated temperature of 600 °C. This specific biochar was manufactured by Hubei Jinzhi Eco-Energy Co., Ltd., situated in Xiaogan City, China. The organic matter biochar used in this study had a pH of 9.42 and exhibited the following elemental composition: nitrogen at 0.74%, carbon at 47.14%, hydrogen at 1.62%, oxygen at 11.85%, phosphorus at 0.32%, potassium at 18.90%, and ash at 19.43%. The pelletized materials, including talc and attapulgite, were procured from Qingdao Ruihua Agricultural Technology Co., Ltd. in Qingdao, China, alongside a model RH-325 small coating machine.

### 2.2. Seed Coating Method

**Figure 1:**
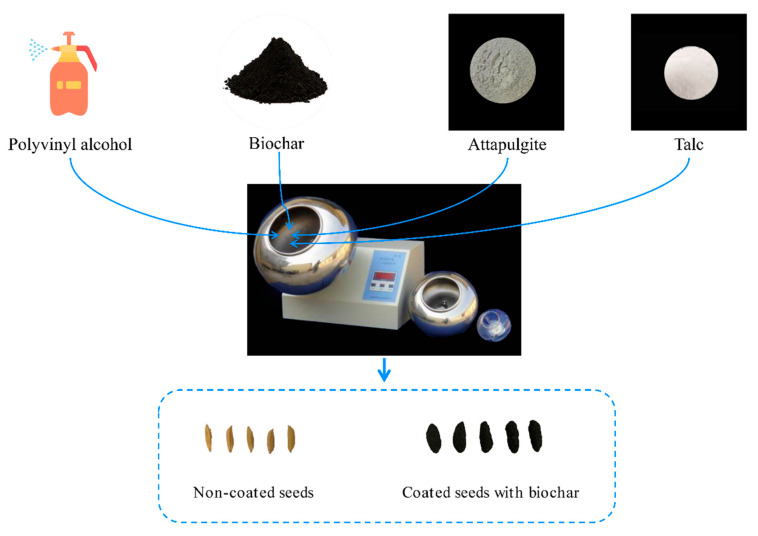
Flow chart of rice seed coating with biochar.

In the seed coating process, the ratio of talc to attapulgite was set at 5:2 (*w*/*w*) [15]. The biochar content in the coating agents varied as follows: 20% (BC20), 30% (BC30), 40% (BC40), 50% (BC50), and 60% (BC60). The mass ratio of seeds to coating agent was 1:2, as recommended. The specific details of seed coating followed the method of Zhang et al. [11]. Briefly, coating agents, including biochar, talc, and attapulgite, were evenly mixed into a seed coating formulation for use (Figure 1). Naked seeds were placed in a rotating cylindrical drum set at 200 rpm. An adhesive agent (polyvinyl alcohol) was then injected for 8 s. Next, half of the coating agents were gradually added into the rotating drum. Simultaneously, the adhesive agent was injected for 1 min and the previous step was repeated. Finally, the drum continued rotating for 2 min to ensure even distribution of the coating agents onto the seeds. The coated seeds were then air-dried at 25 °C.

### 2.3. Seed Germination Assay

This experiment utilized a completely randomized block design within an HP250GS-C artificial climate chamber manufactured by Ningbo Southeast Instrument Co., Ltd. (Ningbo, China). Specific treatments included non-coated seeds (CK) and coated seeds with different contents of biochar (BC20, BC30, BC40, BC50, and BC60). In germination tests, 40 seeds from each variety were planted in germination boxes (12.0 × 12.0 × 6.0 cm) filled with 500 g of air-dried soil. Starting at the time of seed sowing, the soil moisture content was meticulously regulated to the field holding capacity using the weighing method. The soil for the germination box has a yellowish-brown sandy loam texture, containing 5.2% clay, 27.1% silt, and 67.7% sand. The soil exhibited a pH of 6.47, with the following physicochemical properties: organic substance at 16.5 g kg^−1^, nitrate-nitrogen at 23.8 mg kg^−1^, ammonium nitrogen at 24.6 mg kg^−1^, available phosphorus at 14.7 mg kg^−1^, and available potassium at 179.6 mg kg^−1^. Three replicates for each treatment were placed in the growth chamber with a rotation cycle of 12 h light (8000 lx) at 30 °C and 12 h darkness at 25 °C. Following sowing, we diligently recorded the daily count of emerging seeds until it reached a steady state. Seedlings were determined on the ninth day after sowing (DAS).

### 2.4. Determination of Physical Properties of Coated Seeds

#### 2.4.1. Coating Material Loss Test

Three replications of 5.0 g of coated seed from each coating formulation were evaluated using a Model 8411 electric shaking sieve machine (Hangzhou Tongqi Instrument Co., Ltd., Hangzhou, China). Treated seeds were weighed and placed in the standard sieve, No. 25 (0.71 mm opening), to shake for 2 min and then weighed again [16]. Weight loss of coating material was computed by the equation below:

$$Weight\;loss\;(\%)=\frac{100\times {(w}_{1}-w_{2})}{w_{1}}.$$

where *w*_1_ represents the weight of coated seeds and *w*_2_ represents the weight of coated seeds after shaking.

#### 2.4.2. Compressive Strength Test

The compressive strength of a single seed was measured with a precision instrument, a TMS-Pro texture analyzer (Food Technology Corporation, Sterling, VA, USA). Ten coated seeds were randomly chosen from batches of various coating treatments to determine seed surface compressive strength (Force N) [17].

#### 2.4.3. Disintegration Time Test

The disintegration time of different coating formulations can be estimated by the hydration test. Specifically, thirty coated seeds with three replications were put in 20 mL of distilled water to measure disintegration time. The time started from the disintegration of the first seed until the complete disintegration of all seeds, recorded in minutes [16].

### 2.5. Germination and Seedling Attributes

Ten seedlings of two varieties were sampled to measure their shoot length and root length, followed by separating the seedlings into shoots and roots. Subsequently, the roots were scanned by an Epson V800 scanner (Epson Seiko Epson Corporation, Nagano Prefecture, Suwa, Japan). We employed WinRHIZO 2017a software, developed by Regent Instruments in Quebec City, QC, Canada, for the comprehensive analysis of total root length, root surface area, average root diameter, and root volume, as well as the count of root tips. The seedlings were then oven-dried at 75 °C to determine the shoot and root dry weight (DW), respectively. The specific root length (SRL, cm mg^−1^) was calculated by dividing the total root length by the root DW per plant. Other emergence and seedlings parameters were calculated according to the formulae provided by Zhang et al. [11]:

$$Emergence\;rate=\frac{100\times N}{n}.$$

Here, *N* represents the count of normally emerged seedlings, and *n* is the total number of seeds subjected to testing.

$$Emergence\;index=\sum \frac{n}{D}$$

Here, *n* represents the count of emerged seedlings on a specific day, and *D* denotes the corresponding day number.

$$Mean\;emergence\;time=\frac{\sum \left(D\times n\right)}{\sum n}.$$

Here, *n* represents the count of emerged seedlings on day *D*, and *D* denotes the number of days recorded from the initiation of emergence.

$$E50=\frac{t_{i}+(\left(N/2\right)-n_{i})(t_{j}-t_{i})}{n_{j}-n_{i}}.$$

Here, *N* is the final number of emerged seeds and *n_i_* and *n_j_* are the cumulative numbers of seeds emerged by adjacent counts at times *t_i_* and *t_j_* when *n_i_* < *N*/2 < *n_j_*.

Seedling vigor index-I = emergence rate × (shoot length + root length) in cm per plant.

Seedling vigor index-II = emergence rate × (shoot DW + root DW) in mg per plant.

### 2.6. Seed Water Absorption

Fifty seeds were randomly selected from two varieties with three replications and weighed on an electronic balance [18]. Non-coated and coated seeds were placed in the germination box (12.0 cm × 12.0 cm × 6.0 cm) with two layers of filter paper, moistened with 10 mL of distilled water, and then immediately transferred to the growth chambers and maintained at 25 °C in darkness. Uncoated and coated seeds were weighed every 3 h until 24 h. The water uptake of non-coated seeds and coated seeds was calculated as:

$$Water\;uptake\;(\%)=\frac{100\times {(w}_{1}-w_{0})}{w_{0}}.$$

where *w*_0_ is the initial weight before imbibition and *w*_1_ is weight of imbibed seeds.

### 2.7. Respiration Rate, α-Amylase Activity, Soluble Sugar, and Soluble Protein

Fifty seeds of two varieties were germinated on two layers of filters in a germination box. After adding 10 mL of distilled water, all boxes were placed in a growth chamber under the same culture conditions as the seed germination assay. Samples were taken from three replicates at 2 and 4 days after sowing (DAS) to measure the respiration rate, α-Amylase activity, soluble sugar, and soluble protein. The respiration rate was estimated according to the method reported by Hussain et al. [19]. Briefly, rice seeds or seedlings of naked seeds and coated seeds (approximately 2 g) from each material were transferred to a 0.5 L glass bottle, which was connected to a closed-circuit system. The CO_2_ concentration in this bottle was recorded every 2 min.

The concentrations of α-Amylase activity, total soluble sugar, and total soluble protein content in germinated seeds on the second and fourth days after sowing were determined following the protocol provided by Nanjing Jiancheng Bioengineering Institute, Nanjing, China [11]. For the assessment of amylase activity and total soluble protein content, 0.2 g of germinated seeds per genotype were weighed, transferred to 1.8 mL of distilled water, and thoroughly mixed. The crude extract was obtained after cryogenic grinding at 4 °C for 4 min, followed by centrifugation at 10,000 rpm for 10 min. Similarly, the homogenized solution of each treatment was heated continuously in a water bath at 100 °C for 10 min and then cooled. After centrifugation at 10,000 rpm for 10 min, the total soluble sugar concentration in the resulting supernatant was quantified following the instructions of the “Soluble Sugar Assay Kit”.

### 2.8. Statistical Analysis

The statistical analysis was performed using Statistix 9.0 software (Analytical Software, Mckinney, TX, USA). Treatment means were compared using the Least Significant Difference (LSD) test at a significance level of 5%.

## 3. Results

### 3.1. Effects of Biochar Coating on Seed Physical Properties

**Figure 2:**
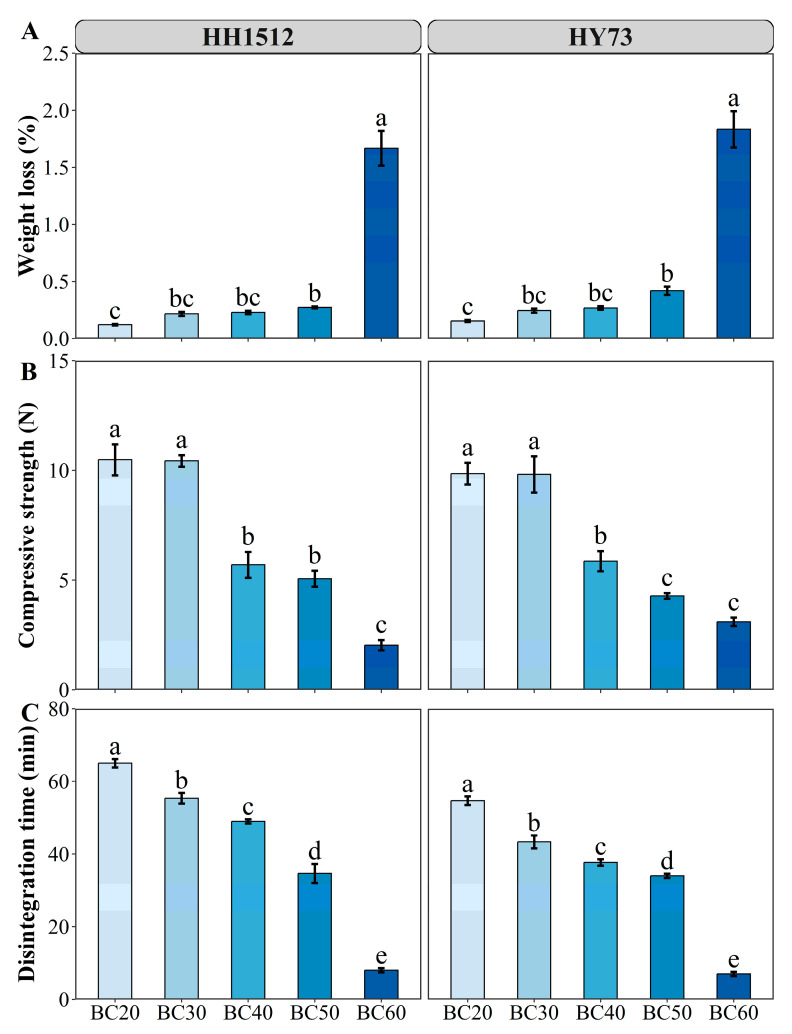
Effects of seed coating with biochar on (**A**) weight loss, (**B**) compressive strength, and (**C**) disintegration time. Varied letters denote statistically significant differences between treatments at *p* < 0.05, as determined by the LSD test. Error bars represent the standard error of three replicates. HY73: Hanyou73. HH1512: Huhan1512. BC: biochar seed coating; the number after BC indicates the percentage of BC in the coating formulation.

In this study, we measured the physical properties of various seed coating formulations, including mechanical, texture, and hydration analyses (Figure 2). The weight loss of coated seeds did not significantly differ between the two crops when the biochar ratio increased from 20% to 50%. However, it significantly increased when the biochar ratio rose to 60% (Figure 2A). When the proportion of biochar in coated seeds reached 60%, the weight loss in HH1512 and HY73 increased from 0.12% to 1.67% and from 0.15% to 1.83%, respectively.

For both varieties, the compressive strength of the coated seeds showed a declining trend with the increasing proportion of biochar in the coating blend (Figure 2B). Specifically, for HH1512, the average force (N) decreased from 10.4 to 2.0 N as the biochar content increased from 30% to 60%, representing a decrease of approximately 80.8%. Similarly, for HY73, the average force decreased from 9.87 to 3.13 N, representing a reduction of about 68.0%.

The proportion of biochar in the seed coating formulations significantly affected disintegration time (Figure 2C). A higher proportion of biochar in the coating blend for HH1512 seeds decreased the disintegration time from 65 to 8 min. This pattern was also observed for HY73 seeds; as the ratio of biochar increased from 20% to 60%, the disintegration time significantly decreased from 55 to 7 min. In addition, for both varieties of coated seeds, the 20–50% biochar treatments resulted in a gradual decrease in degradation time to 30 min, while the 60% biochar decreased sharply to 7–8 min.

### 3.2. Effects of Biochar Coating on Emergence Traits

**Figure 3:**
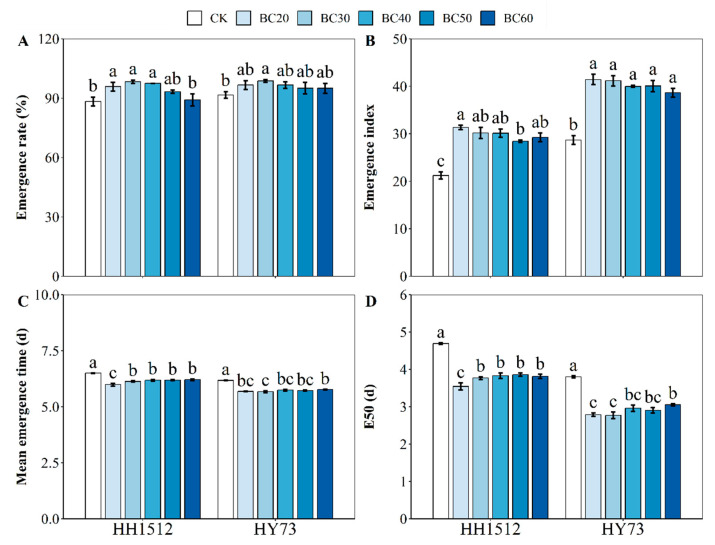
Effects of seed coating with biochar on (**A**) emergence rate, (**B**) emergence index, (**C**) mean emergence time, and (**D**) E50. Varied letters denote statistically significant differences between treatments at *p* < 0.05, as determined by the LSD test. Error bars represent the standard error of three replicates. HY73: Hanyou73. HH1512: Huhan1512. BC: biochar seed coating; the number after BC indicates the percentage of BC in the coating formulation.

Biochar coating improved both the emergence rate and emergence index while reducing mean emergence time (MET) and E50 in both cultivars (Figure 3). The effect of seed coating on emergence rate showed a trend of first increasing and then decreasing with an increase in biochar content for both genotypes (Figure 3A). For HH1512, treatments BC20, BC30, and BC40 achieved an emergence rate of 95%, significantly increasing by 8.5%, 11.3%, and 10.4%, respectively, compared with untreated seeds (88%). The emergence rate for HY73 under treatments BC20, BC30, BC40, BC50, and BC60 reached or exceeded 95%. The optimal concentration of biochar was found to be at a level of 30%, which significantly increased the emergence rate by 7.7% relative to non-coated seeds (Figure 3A). Compared to control conditions, seed coating with biochar at ratios ranging from 20% to 60% significantly improved the emergence index by between 34.1% and 47.7% and between 82.3% and 95.4% for HH1512 and HY73, respectively (Figure 3B). In addition, a 20–40% biochar coating was the most effective in terms of emergence index, and HY73 was superior to HH1512. All biochar coating treatments significantly decreased MET and E50 compared with controls in both lines tested (Figure 3C,D). Moreover, MET and E50 values for biochar coatings at levels between 20% and 30% were lower than those for other coating treatments. This reduced MET by between 0.37 and 0.51 days and E50 by between 0.92 and 1.15 days for HH1512. Similarly, it reduced MET by between 0.49 and 0.51 days and E50 by between 1.01 and 1.03 days for HY73.

### 3.3. Effects of Biochar Coating on Seedling Establishment

**Figure 4:**
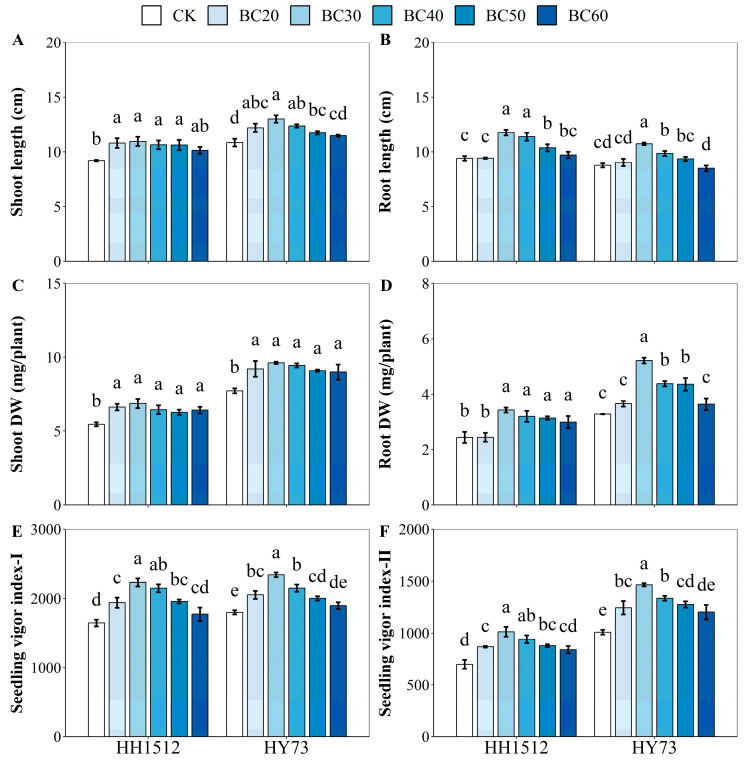
Effects of seed coating with biochar on (**A**) shoot length, (**B**) root length, (**C**) shoot DW, (**D**) root DW, (**E**) seedling vigor index-I, and (**F**) seedling vigor index-II. Varied letters denote statistically significant differences between treatments at *p* < 0.05, as determined by the LSD test. Error bars represent the standard error of three replicates. HY73: Hanyou73. HH1512: Huhan1512. BC: biochar seed coating; the number after BC indicates the percentage of BC in the coating formulation. DW: dry weight.

**Figure 5:**
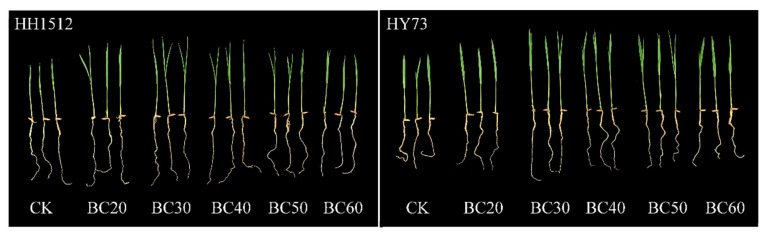
Effects of seed coating with biochar on phenotypic traits of WDR seedling. HY73: Hanyou73. HH1512: Huhan1512. The number after BC indicates the percentage of BC in the coating formulation.

For both cultivars, the morphology and seedling vigor index of treated seedlings were higher than those of the control (Figure 4 and Figure 5). In both cultivars, the shoot growth of biochar-treated seedlings was significantly longer than that of untreated seedlings, increasing by between 15.4% and 19.0% for HH1512 and between 8.3% and 19.9% for HY73, except for BC60 (Figure 4A and Figure 5). Compared to an untreated control, a seed coating with BC30, BC40, and BC50 significantly promoted root length and root DW; the increased range for HH1512 was between 10.1% and 24.9% and between 28.7% and 40.7%, respectively, while for HY73 it was between 6.7% and 22.4% and between 32.8% and 58.9%, respectively (Figure 4B,D). The dry weight of treated shoots from both cultivars was significantly higher than that of the control, boosting shoot DW by a range of between 14.7% and 25.6% and between 16.7% and 24.6% for HH1512 and HY73, respectively (Figure 4C). All coating treatments with biochar significantly raised seedling vigor index-I (SVI-I) and seedling vigor index-II (SVI-II) compared to an uncoated control for both varieties (Figure 4E,F), except for BC60. Averaging across cultivars, SVI-I and SVI-II values for seed coatings with biochar from 20% to 50% improved by between 15.2% and 33.1% and between 23.8% and 45.0%, respectively. In HH1512, seed coating with BC30 enhanced shoot length, root length, shoot DW, root DW, SVI-I, and SVI-II by 19.0%, 24.9%, 25.6%, 40.7%, 35.8%, and 44.8%, respectively, compared to control; the respective increases for HY73 were 19.9%, 22.4%, 24.6%, 58.9%, 30.4%, and 45.3%, respectively (Figure 4 and Figure 5). Notably, the most pronounced effects were recorded at a proportion of 30% among these biochar coating treatments, irrespective of variety.

### 3.4. Effects of Biochar Coating on Seedling Root Architecture

**Figure 6:**
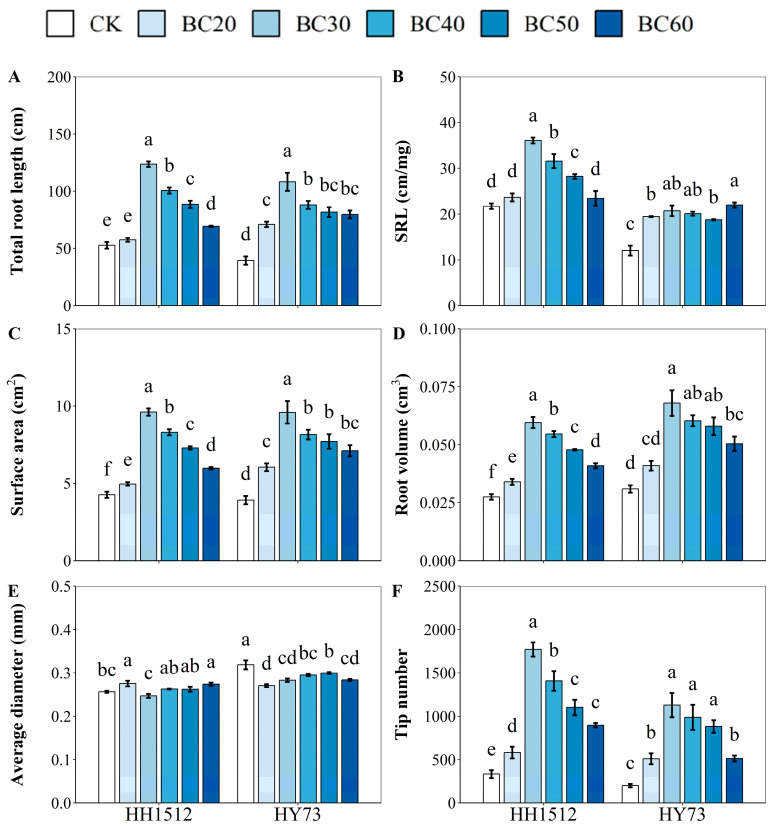
Effects of seed coating with biochar on (**A**) total root length, (**B**) specific root length (SRL), (**C**) surface area, (**D**) root volume, (**E**) average diameter, and (**F**) tip number. Varied letters denote statistically significant differences between treatments at *p* < 0.05, as determined by the LSD test. Error bars represent the standard error of three replicates. HY73: Hanyou73. HH1512: Huhan1512. BC: biochar seed coating; the number after BC indicates the percentage of BC in the coating formulation.

All biochar coating treatments for both cultivars significantly promoted the development of the root system, including surface area (SA), root volume (RV), and tip number, except for the RV of HY73 with the BC20 treatment (Figure 6). For HH1512, biochar coating at four concentration levels (30%, 40%, 50%, and 60%) significantly enhanced total root length (TRL) and specific root length (SRL), except for BC60, compared to the control treatment. There were significant differences among the four treatments (Figure 6A,B). The four coating treatments with biochar enhanced TRL by 134.5%, 90.7%, 68.0%, and 31.7%, and SRL by 66.1%, 45.3%, 29.9%, and 7.8%, respectively. All five concentrations of biochar coating significantly increased SA, RV, and tips when compared to no biochar coating, and there were significant variations between these five concentrations (Figure 6C,D,F). Relative to non-coated seeds, the biochar coating treatments promoted SA, RV, and tips by an average of 83.1%, 84.8%, and 288.5%, respectively. Average diameter (AD) was improved under coating with biochar at a ratio of 20% and 60% by 7.4% and 6.7%, respectively, and reached a significant level compared to the control (Figure 6E). Likewise, biochar coating with a content of 30% was the most effective treatment, which enhanced TRL, SRL, SA, RV, and tips by 134.5%, 66.1%, 125.9%, 117.0%, and 431.5%.

For HY73, all coating treatments with biochar significantly improved TRL, SRL, SA, RV, and tips, except for RV at a biochar content level of 20%. When compared to the uncoated control, the coating treatment boosted TRL, SRL, SA, RV, and tips by an average of 117.1%, 67.8%, 97.4%, 79.7%, and 300.6% across all biochar coating treatments (Figure 6A–D,F). However, AD was significantly reduced by seed coating with biochar from a content level of 20% to 60%, resulting in a reduction range of 5.9–11.1% relative to the non-coated treatment (Figure 6E). The finer the roots of the seedling, the easier it is to access moisture in the small pores of the soil. Among various coating treatments, the BC30 treatment showed the best performance in terms of seedling root morphology. Biochar coating with a proportion of 30% increased TRL, SRL, SA, RV, and tips by 173.9%, 72.0%, 145.3%, 119.9%, and 461.6%, respectively, while lowering AD by 11.2%. Based on these experimental results, highly significant differences in seed coating treatments were observed in response to emergence traits and seedling growth at a biochar content level of 30%. Therefore, biochar coating with a ratio of 30% (BC30) was adopted for further research.

### 3.5. Biochar Coating Increased Seed Moisture Absorption

**Figure 7:**
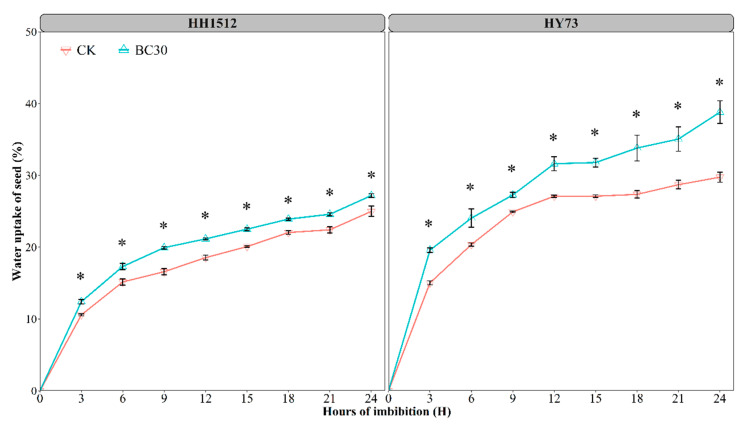
Effects of the optimal biochar coating on the water uptake of seed. The asterisks denote statistically significant differences between treatments at *p* < 0.05, as determined by the LSD test. Error bars represent the standard error of three replicates. HY73: Hanyou73. HH1512: Huhan1512. CK: non-coated seeds. BC30: seed coating with biochar at 30% content.

Changes in seed water uptake over 24 h for both genotypes were recorded and are presented in Figure 7. When compared to uncoated seeds, seeds coated with biochar showed significantly higher water absorption in both varieties. Moreover, HY73 showed faster and higher quantities of water absorption than HH1512 at the same imbibing time, regardless of different seed coatings. Biochar coating increased the water absorption of HH1512 seeds by 17.3%, 14.1%, 20.1%, 14.1%, 12.1%, 8.5%, 9.7%, and 8.7% at 3, 6, 9, 12, 15, 18, 21, and 24 h after imbibition, respectively, as compared to the control. The corresponding increases for HY73 were 30.2%, 18.1%, 8.9%, 16.7%, 17.3%, 23.6%, 22.1%, and 30.4%, respectively (Figure 7).

### 3.6. Biochar Coating Improved Respiration Rate, α-Amylase Activity, Soluble Sugar and, Soluble Protein

**Figure 8:**
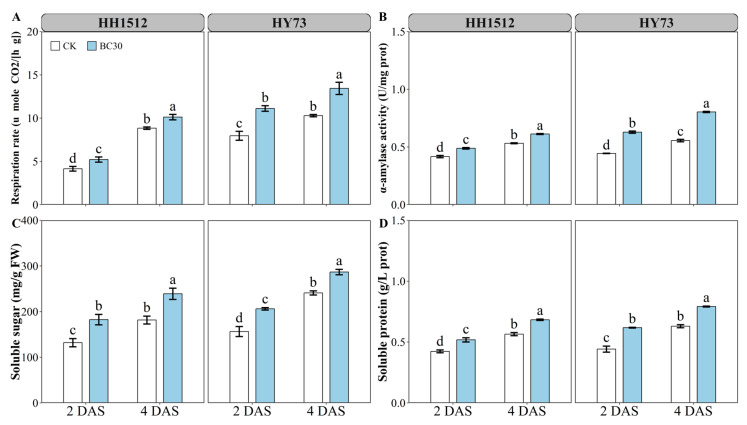
Effects of the optimal biochar coating on (**A**) respiration rate, (**B**) α-amylase activity, (**C**) soluble sugar, and (**D**) soluble protein. Varied letters denote statistically significant differences between treatments at *p* < 0.05, as determined by the LSD test. Error bars represent the standard error of three replicates. HY73: Hanyou73. HH1512: Huhan1512. CK: non-coated seeds. BC30: seed coating with biochar at 30% content.

In the present study, a 30% biochar coating significantly promoted the respiration rate, α-amylase activity, total soluble sugar, and total soluble protein contents of germinated rice seeds at 2 and 4 days after sowing (DAS) (Figure 8). Compared to the control, the respiration rate of HH1512 under seed coating with biochar increased by 25.7% and 14.4% at 2 and 4 DAS, respectively. Similarly, the enhancement for HY73 was 39.6% at 2 DAS and 30.6% at 4 DAS (Figure 8A). At 2 DAS, coating with biochar elevated α-amylase activities by 17.3% and 41.3% in HH1512 and HY73, respectively, relative to controls; at 4 DAS, seed coating for HH1512 and HY73 improved α-amylase activities by 15.0% and 44.6%, respectively (Figure 8B). Biochar coating increased the soluble sugar and soluble protein of HH1512 seeds by 38.2% and 22.2% at 2 DAS, and by 31.6% and 20.9% at 4 DAS, respectively, compared to the non-coated treatment. Similarly, the increase in soluble sugar and soluble protein for HY73 was observed at 2 DAS at levels of 31.8% and 39.8%, and at 4 DAS at levels of 18.9% and 25.7%, respectively (Figure 8C,D).

## 4. Discussion

The integrity of coated seeds is a crucial trait as it is linked to seed germination and the potential for breakage during handling, distribution, and sowing. On the one hand, materials that are too hard or impervious to the outer layer of coated seeds can prevent or inhibit germination. The surface materials of pelleted seeds must have better mechanical properties to ensure that they do not disintegrate or break prior to planting. Therefore, it is extremely important to quantitatively evaluate the likelihood of cracks and weight loss that can occur during handling and transportation. To meet environmental safety standards [20], we determined the integrity of seeds coated with favorable carbon-based biochar. The results showed that weight loss was relatively low for both varieties at low biochar content levels (20–50%), however the rate of loss increased dramatically when biochar content exceeded 50% (Figure 2A). This result may be due to the loose and porous structure of biochar particles [21]. The higher the content of loose biochar during seed coating, the more difficult it is to pelletize into clusters and the more likely it is to break up. Our results were similarly confirmed by the fact that the compressive strength of single seeds gradually decreased as the proportion of biochar in the coating formulations of two cultivars increased (Figure 2B). In addition, we found that when the biochar content in the coating formulation was higher than 30%, the compressive strength of the coated seeds showed a steep decline. Nevertheless, the mechanical integrity data of the coating formulations with biochar at a ratio of 20–40% observed in the current study are in accordance with European standards and meet the benchmarks for the safety of dust generation from coated seeds [22].

The wet strength of a seed coating primarily depends on the adhesive properties of ingredients submerged in water. Theoretically, the longer it takes for a coating to decompose when immersed in water, the greater the likelihood that it will impede emergence. A hydration trial was performed to estimate the time required for a coating to disintegrate while immersed in water and to evaluate its potential to retard germination. The present result showed that various proportions of biochar in seed coating blends had a pronounced influence on disintegration time for both genotypes (Figure 2C). In particular, a coating treatment containing 60% biochar took only 7.5 min to completely dissolve in water, averaged across two varieties. These results suggest that the germination characteristics of coated seeds may not be compromised by biochar. The easy degradation of biochar coating formulations while in contact with water may be closely related to its non-binding and water-absorbent properties. Since biochar is a non-adhesive, active material, it is necessary to apply a certain binder to encapsulate the biochar particles; otherwise, they will easily fall apart.

In this study, seed coating with a low ratio biochar (20–40%) improved emergence rate, shoot length, and shoot dry weight from both varieties (Figure 3 and Figure 4), while a slight decrease in the above traits was observed in treatments with higher content levels (above 40%). This indicates that a higher proportion of biochar in seed coating formulations may have an inhibitory effect on seed germination and seedling growth. A possible explanation is the strong alkalinity of biochar particles when dissolved in water. Previous studies have also confirmed our findings that the use of high concentrations of biochar coating had unfavorable effects on seed germination and even impaired germination performance [11,23].

Furthermore, a 30% biochar treatment performed the best, regarding seedling performance, among all seed coating formulations, regardless of whether the variety was HH1512 or HY73 (Figure 3, Figure 4 and Figure 5). This was reflected in an improved emergence rate (9.5%), shoot length (19.5%), shoot DW (25.1%), SVI-I (33.1%), and SVI-II (45.0%) compared to controls, across both rice materials. It has been demonstrated by previous studies that growth-regulating compounds similar to auxin and cytokinin present in biochar particles may stimulate seed germination and seedling development through the generation of strong chemical signals [24]. Additionally, biochar may also improve the opportunity for moisture and nutrient availability around the coated seed due to the physicochemical properties of biochar, such as strong water uptake and rich organic nutrient content [25]. This speculation was confirmed by the fact that biochar-coated seeds in two rice varieties absorbed more water within 24 h of imbibition compared to uncoated seeds (Figure 7). It has been well documented that biochar exhibits strong moisture uptake and retention properties, mainly attributed to its large, specific surface area and highly porous structure [12,26]. A similar finding was reported by Adelabu and Franke [13], where biochar coating blends had a substantial and positive impact on germination, seedling elongation, and fresh biomass in okra. They hypothesized that since biochar is a carbonaceous organic matter and a source of abundant nutrients, it could result in an increase in plant growth and biomass accumulation by supplying rich nutrition to the seedling embryo during emergence.

Biochar used for seed coating is applied to the soil or root surface along with the seed, which inevitably affects the root morphology of plant seedlings. Therefore, it is critical to study the response of root morphology to the application of different levels of biochar. In this study, appropriate levels of biochar (30%–50%) significantly improved root system architecture in two rice varieties compared to the control (Figure 4 and Figure 6). However, no significant variation was noted in either low (20%) or high (60%) biochar treatments, such as root length, root DW, and SRL. The 30% biochar treatment was the most effective for increasing root morphological traits in both rice cultivars. These results revealed that a suitable proportion of biochar was beneficial for optimizing the root structure at the seedling stage, which could be attributed to the excellent properties of biochar. Specifically, biochar directly provided the root system with a loose and porous growth environment that retained water and nutrients. Extensive prior research has consistently documented that biochar, owing to its elevated organic carbon content and substantial porosity, fosters a habitat conducive to microbial proliferation and life. Thus, biochar may stimulate the vitality of growth-promoting bacteria around seeds, which in turn improves root development [7,8,9].

In this study, α-amylase activity and soluble sugar levels in germinated seeds from both genotypes increased by 29.3% and 35.0% at 2 DAS and by 29.9% and 25.3% at 4 DAS, respectively, across averaged varieties in response to optimal biochar treatment (BC30) (Figure 8B,C). These findings demonstrated that biochar coating resulted in higher starch metabolism in seeds, which led to greater activity of α-amylase and thereby promoted the breakdown of starch into soluble sugars for better growth and maintenance processes. Previously, activated starch metabolism has been strongly associated with respiration rate [19]. The present findings showed that seeds of two rice varieties coated with biochar had higher respiration rates (Figure 8A), which was consistent with enhanced starch metabolism and vigorous seedling growth (Figure 3). Stronger respiration rates in coated rice seedlings produced more ATP, which in turn accelerated emergence and seedling growth. Researchers found that the rapid increase in respiration rate coincided with the emergence of the radicle [27]. In addition, it was noted that the increase in respiration rate was the result of the activation of α-amylase [28], suggesting that the increase in respiration rate in coated seedlings is closely related to the activation of starch metabolism and subsequent seedling development. Soluble proteins can provide seedlings with a food supply and specific proteins, such as cell membrane transporter proteins, during seed storage protein degradation [29]. The current results demonstrated that biochar seed coating induced a relatively high accumulation of soluble proteins compared to the untreated control (Figure 8D). High concentrations of soluble proteins can provide the substrate needed to produce the energy required for seedling development.

## 5. Conclusions

In summary, biochar applied as a seed coating material has the potential to effectively maximize emergence traits and seedling establishment in both WDR varieties. The current study clearly demonstrated that biochar, a carbonaceous organic substance, acts as a desirable active ingredient in coating formulations to stimulate seed germination and crop stand. On the basis of the physical properties and seedling performance of coated seeds, a 30% biochar coating was screened as the optimal choice among all seed coating blends for two genotypes. The promoted emergence and vigorous seedling growth induced by the biochar-coated treatment were correlated with an increase in water uptake by seeds, higher starch metabolism and respiration rate, and improved accumulation of soluble protein in these seeds. However, future research on biochar-based seed coating could be carried out to develop innovative and efficient seed coating formulations by employing different biochar feedstocks, coating compositions, and coating technologies. In addition, further work at the metagenomic level of soil microorganisms is inevitable to explore the interacting mechanisms of plant-microbe-soil induced by seed coating with biochar.

## Data Availability

Data are contained within the article.
